# The Colombo Twin and Singleton Follow-up Study: a population based twin study of psychiatric disorders and metabolic syndrome in Sri Lanka

**DOI:** 10.1186/s12889-017-4992-2

**Published:** 2018-01-17

**Authors:** Kaushalya Jayaweera, Lisa Aschan, Gayani Pannala, Anushka Adikari, Nicholas Glozier, Khalida Ismail, Carmine M. Pariante, Fruhling Rijsdijk, Sisira Siribaddana, Helena M. S. Zavos, Patricia A. Zunszain, Athula Sumathipala, Matthew Hotopf

**Affiliations:** 1 Institute for Research and Development, Colombo, Sri Lanka; 2 Psychological Medicine Department, Institute of Psychiatry, Psychology, and Neuroscience, King’s College London, London, UK; 3 NIHR Biomedical Research Centre for Mental Health at the South London and Maudsley NHS Foundation Trust, King’s College London, London, UK; 4 Brain and Mind Centre, University of Sydney, Sydney, Australia; 5 Stress, Psychiatry and Immunology Laboratory, Institute of Psychiatry, Psychology & Neuroscience, King’s College London, London, UK; 6 Social Genetic and Developmental Research Centre, Institute of Psychiatry, Psychology & Neuroscience, King’s College London, London, UK; 7 Department of Medicine, University of Rajarata, Anuradhapura, Sri Lanka; 8 Department of Psychology, Institute of Psychiatry, Psychology & Neuroscience, King’s College London, London, UK; 9 Research Institute for Primary Care & Health Sciences, Faculty of Medicine & Health Sciences, Keele University, Keele, UK

## Abstract

**Background:**

The disease burden related to mental disorders and metabolic syndrome is growing in low-and middle-income countries (LMIC). The Colombo Twin and Singleton Study (COTASS) is a population-based sample of twins and singletons in Colombo, Sri Lanka. Here we present prevalence estimates for metabolic syndrome (metS) and mental disorders from a follow-up (COTASS-2) of the original study (COTASS-1), which was a mental health survey.

**Methods:**

In COTASS-2, participants completed structured interviews, anthropometric measures and provided fasting blood and urine samples. Depressive disorder, depressive symptoms, anxiety symptoms, post-traumatic stress disorder (PTSD) and hazardous alcohol use were ascertained with structured psychiatric screens (Composite International Diagnostic Interview (CIDI), Beck Depression Inventory (BDI-II), Generalised Anxiety Disorder Questionnaire (GAD-7), PTSD Checklist – Civilian Version (PCL-C), and Alcohol Use Disorders Identification Test (AUDIT)). We defined metS according to the International Diabetes Federation (IDF) criteria and the revised National Cholesterol Education Programme Adult Treatment Panel (NCEP ATP III) criteria. We estimated the prevalence of psychiatric disorders and metS and metS components, and associations with gender, education and age.

**Results:**

Two thousand nine hundred thirty-four twins and 1035 singletons were followed up from COTASS-1 (83.4 and 61.8% participation rate, respectively). Prevalence estimates for depressive disorder (CIDI), depressive symptoms (BDI ≥ 16), anxiety symptoms (GAD-7 ≥ 10) and PTSD (PCL-C DSM criteria) were 3.8, 5.9, 3.6, and 4.5% respectively for twins and 3.9, 9.8, 5.1 and 5.4% for singletons. 28.1 and 30.9% of male twins and singletons respectively reported hazardous alcohol use. Approximately one third met the metS criteria (IDF: 27.4% twins, 44.6% singletons; NCEP ATP III: 30.6% twins, 48.6% singletons). The most prevalent components were central obesity (59.2% twins, 71.2% singletons) and raised fasting blood glucose or diabetes (38.2% twins, 56.7% singletons).

**Conclusion:**

MetS was highly prevalent in twins, and especially high in singletons, whereas the prevalence of mental disorders was low, but consistent with local estimates. The high levels of raised fasting plasma glucose and central obesity were particularly concerning, and warrant national diabetes prevention programmes.

**Electronic supplementary material:**

The online version of this article (doi:10.1186/s12889-017-4992-2) contains supplementary material, which is available to authorized users.

## Background

Low and middle income countries (LMIC) globally have undergone rapid urbanisation, and changes in demography and health behaviours [1, 2]. Whilst life expectancy at birth for many LMIC has improved, disease burden from non-communicable diseases, and years lived with disability have risen [3, 4]. In 2012, 82% of all global premature deaths attributable to non-communicable diseases occurred in LMICs [2]. The global burden of mental disorders, particularly depression, is also forecast to rise and depression is strongly associated with many non-communicable diseases [5]. In Sri Lanka, the setting for the present study, coronary heart disease is the leading cause of mortality, one in ten in the population has diabetes, and there has been an exponential increase in hospitalisations due to these diseases [6–8]. High prevalence estimates of their risk factors, including hypertension (18–20%), dysglycaemia (14–20%) and obesity (9–36%) have been reported [7, 9–11].

MetS represents a cluster of metabolic abnormalities which indicate an elevated risk of future development of type II diabetes and cardiovascular disease (CVD) [12–17]. Its status as a syndrome, and its predictive validity as a *specific* risk factor for type 2 diabetes and CVD (over and above its constituent parts), are debated and some have called into question the usefulness of metS as a specific entity [18]. The confidence in its nosological status could be improved if it could be demonstrated that the clustered phenotypes are driven by the same underlying genes. This may be tested using multivariable behavioural genetics, which permits a better understanding of the genetic architecture of the syndrome would strengthen the biological plausibility of metS. For this reason, we conducted a study of cardiometabolic risk using a sample of twins and singletons in Colombo, Sri Lanka; a study design which allows for these types of analyses.

Studying cardiometabolic risk in a South Asian population in a genetically sensitive design is of particular interest given suggestions that the elevated incidence of cardiovascular disease and diabetes in South Asian populations may be partially explained by mechanisms involving gene-environment interactions [19]. Such heritable adaptations may include those which improve resilience to prolonged periods of food shortage (the so-called “thrifty genotype”), but at times of excess lead to greater susceptibility of developing obesity and other metS phenotypes [20, 21]. This may mean that metS in South Asian populations is different in terms both of phenotypic associations and genetic architecture compared with Western populations.

This paper describes the Colombo Twin and Singleton Follow-up Study (COTASS-2) – a population based study of twins and singletons from the Colombo District, Sri Lanka. COTASS-2 explores mental health and cardiovascular risk factors, and the role of genetic and environmental influences on their variance and covariance. The specific aims of COTASS-2 were to: (1) estimate the (genetic) stability of depression in a south Asian population; (2) describe the prevalence of the component phenotypes which make up “metS” in Sri Lanka; (3) explore the genetic architecture of metS phenotypes, and estimate the extent to which phenotypic associations are explained by shared genetic and environmental effects and (4) investigate the aetiological overlap between depression and the component phenotypes of metS.

In the present paper we describe the methods of COTASS-2 and present prevalence estimates of metS, its components, and mental disorders, separately for twins and singletons.

## Methods

### Setting

Colombo District has a population of 2.32 m, composed of multiple ethnic groups, including Sinhalese (76.5%), Tamils (11.2%) and Moors (10.7%). It is mainly classified as urban (77.6%) and includes the capital city of Sri Lanka, Colombo, but the wider district also includes rural areas [22]. Typical of many urban regions, the district attracts a high level of immigration, of which a substantial minority have been displaced as a result of conflict [23]. The population is characterised by great socio-economic diversity in terms of education, employment, and occupational social class. Since data collection ended for the original COTASS study in 2007, the three decade-long civil conflict in Sri Lanka came to an end.

### Description of COTASS-2

COTASS-2 took place between 2012 and 2015, and is a follow-up study of the Colombo Twin and Singleton Study (COTASS-1), conducted in 2005–2007 [24]. COTASS-1 focussed on mental health, and achieved a 91% participation rate for a carefully ascertained population of twins residing in Colombo District, and an 87% participation rate for singletons recruited from the same area. COTASS-2 consisted of three components: an interview component, collection of anthropometric data, and biosample (blood and urine) collection for clinical investigations and biobanking. Two sub-studies examining the autonomic nervous system using heart rate variability measures, and sleep and physical activity using actigraphy were nested within COTASS-2. The study received ethical approval from Psychiatry, Nursing & Midwifery Research Ethics Subcommittee, King’s College London, UK (reference number: PNM/10/11-124), and the Faculty of Medical Sciences University of Sri Jayewardenepura Ethical Review Committee (USJP ERC) (reference number: 596/11).

### Participant tracing and data collection

#### Participant tracing and recruitment

The recruitment process of the original COTASS-1 sample is described in Siribaddana et al. [24]. In recruiting for COTASS-2, COTASS-1 participants were sent invitation letters, and trained field research assistants (FRAs) traced participants by telephone and home visits. Written informed consent was obtained for each study component that they opted to partake in. Participants unable to understand the consent process or the questionnaires due to language barriers or apparent cognitive impairments were excluded. Participants successfully completing one or more study components were offered 750 LKR (approximately £3.50 GBP) to compensate for time and inconvenience.

#### Interview data collection

Fifteen FRAs were involved in recruitment and questionnaire data collection. All FRAs had at minimum high school qualifications. They received training on basic research methodology and research ethics; collecting informed consent, and conducting the paper and pen based questionnaire interviews. Interviews lasted 1–2 h and were typically conducted in participants’ homes. Quality checks were performed throughout data collection. Study coordinators performed random unannounced spot checks while FRAs conducted interviews. The study coordinators and the data entry team checked questionnaires thoroughly for errors and inconsistencies before accepting completed questionnaire booklets. A sub-sample of randomly selected participants were phoned to confirm that the collected questionnaire data were accurate. An experienced data entry team entered data into a database using SPSS version 13 [25].

#### Interview measures

Interviews involved 19 scales and checklists measuring sociodemographic characteristic, health and functioning measures including structured assessments of psychiatric disorders, health behaviours, stressful life events and social support, and zygosity (see Additional file 1: Table S1).

##### Sociodemographic characteristics

Sociodemographic information was collected through adapted Sri Lankan census measures [22], including gender, age, ethnicity, occupation, education, household composition and housing quality.

##### Health and functioning measures

Structured symptom screens measured several psychiatric disorders. Section E of the World Health Organization’s Composite International Diagnostic Interview (CIDI) captured probable depression diagnoses over the past year [26], and the Beck Depression Inventory (BDI) captured depressive symptom severity over the past 2 weeks [27]. Post-traumatic stress disorder and anxiety were measured using the PTSD Checklist Civilian version [28] and the Generalised Anxiety Disorder questionnaire [29] respectively. The Bradford Somatic Inventory [30] screened for somatic symptoms while the Chalder Fatigue Scale [31] measured the severity and extent of fatigue. The Dutch Eating Behaviour Questionnaire [32] and the Three-Factor Eating Questionnaire [33] identified eating disorders. The Short Form 36 Health Survey Questionnaire [34] measured general health and wellbeing. Sleep over the month was assessed using the Pittsburgh Sleep Quality Index [35]. A physical illness checklist, developed by the Institute of Research and Development (IRD) Sri Lanka, identified significant current and life-time physical illnesses (requiring medication).

##### Health behaviours

The International Physical Activity Questionnaire [36], an internationally validated scale, captured exercise over the past 7 days. Dietary patterns were measured using a culturally adapted version of the food frequency questionnaire; revised in consultation with local experts [37]. Tobacco and alcohol use were measured using adapted versions of the Tobacco Use questionnaire of the WHO STEPS Instrument [38] and the WHO’s Alcohol Use Disorders Identification Test (AUDIT) [39].

##### Stressful life events and social support

Stressful life events were measured using a culturally adapted version of the list of threatening experiences questionnaire [40]. Social support and psychological wellbeing was measured using a modified version of the multi-dimensional support scale questionnaire [41].

##### Zygosity and closeness-of-twins measures

Zygosity was ascertained in COTASS-1 using a questionnaire measure of similarity [24, 42]. If zygosity was missing in COTASS-1 it was replaced with zygosity information collected using the same questionnaire in COTASS-2 (*n* = 88). The closeness of twins within pairs was measured using items adapted from a study which measured individual differences in personality, ability and interests [43], and another study on psychiatric disorders which measured similarity of environmental experiences of twins [44].

##### Translation and adaptation of new scales for COTASS-2

Many of the questionnaire components had already been translated and validated in COTASS-1 and other projects [24, 45]. The new scales for COTASS-2 were translated by a panel consisting of Sri Lankan health professionals, academics, and a scholar fluent in both Sinhala and English. Translations were not literal, but cross-culturally adapted to Sinhala in wording which best conveyed the same meaning [46]. Interviews were conducted in Sinhala or English depending on participants’ preference.

#### Anthropometric and blood pressure measurements

Twelve FRAs were trained to collect anthropometric and blood pressure measurements according to standard protocols, contemporaneously with the interviews. Anthropometric measures included standing height, sitting height, weight, waist circumference and were measured to the closest 0.1 cm/kg. Leg length was estimated from standing height and sitting height measured using portable stadiometers (Seca, Germany). BMI was calculated from standing height and weight using electronic weighing scales (Seca, Germany). Waist circumference was measured using measuring tapes. Blood pressure was measured using Omron HEM-7200 automatic blood pressure monitors (Omron Healthcare, Japan). Three blood pressure recordings were obtained from after 10 min of rest with 2–3 min intervals between measurements. Quality checks were done by random spot checking, and by the data entry team.

#### Biosample collection

Fasting blood and first morning urine samples were collected during early morning visits to participants’ homes, at the IRD, or specified laboratories. Blood samples were collected using evacuated blood collection tubes (Becton Dickinson, USA). From blood samples the following clinical measures were extracted: fasting blood glucose, HbA1C (high-performance liquid chromatography method), lipid profile, serum glutamic oxaloacetic transaminase (SGOT), serum insulin levels, serum creatinine, and highly sensitive C-reactive protein. Separate blood samples were collected for extracting DNA and serum separation to be stored in a biobank for future genetic analyses. Urine samples provided measures of urine creatinine, urine microalbumin, and urine microalbumin-to-creatinine ratio.

#### Derived measures of metabolic syndrome

We used the most frequently applied metS definitions: the International Federation of Diabetes (IDF) criteria [47], and the revised National Cholesterol Education Programme Adult Treatment Panel (NCEP ATP III) criteria [48]. The revised NCEP ATP III and the IDF criteria share the same components and cut-offs, but are combined differently to indicate metS. These components include: (1) central obesity (waist circumference: ≥90 cm in men, ≥80 cm in women; South Asian population-specific); (2) raised triglycerides (≥1.7 mmol/l); (3) reduced HDL-cholesterol (<1.03 mmol/l in men, <1.29 mmol/l in women); (4) raised blood pressure (systolic ≥130 mmHg, or diastolic ≥85 mmHg, or hypertensive treatment or previously diagnosed hypertension); and (5) raised fasting plasma glucose (≥5.6 mmol/l or previously diagnosed type 2 diabetes). The IDF criteria defines metS as central obesity in combination with any two of the other four components. The revised NCEP ATP III criteria accept the presence of any three components to indicate metS, without considering any component essential.

### Statistical analyses

Percentage prevalence estimates described the sociodemographic and socioeconomic characteristics of the full sample and for twin and singletons separately. To examine possible attritional bias, we used COTASS-1 data to describe differences between participants who were in both study waves, and those who only part-took in COTASS-1. Chi-square tests and t-tests tested distribution differences, and logistic regression controlled for potential confounding factors. Analyses were conducted in Stata 14 [49] and SPSS 13 [25]. Interferential statistics accounted for clustering by twins using the ‘*svy*’ command in Stata.

The prevalence of cardiometabolic risk indicators and mental disorders were estimated and stratified by twin/singleton status, as preliminary analysis indicated that they differed on key demographic variables. Psychiatric disorders included depressive disorder (CIDI), depressive symptoms (BDI-II 16-point cut-off [50]), generalised anxiety disorder (GAD-7 10-point cut-off), hazardous alcohol use (AUDIT 8-point cut-off), and post-traumatic stress disorder (PTSD; PCL-C coded according to DSM criteria). Cardiometabolic risk indicators included metS and all its component parts, and a binary BMI measure applying a South Asian specific cut-off of 23 kg/m^2^ [51]. MetS was only estimated for respondents with complete data for all components. Cross-tabulations described the prevalence distributions of psychiatric disorders and cardiometabolic risk indicators by gender, education, and age groups of 19–34, 35–54, 55+. Separate logistic regression models tested associations with these demographic characteristics, whilst adjusting for gender, education, age (continuous measure when used as covariate), marital status, ethnicity and financial strain.

## Results

### Recruitment

**Fig. 1:**
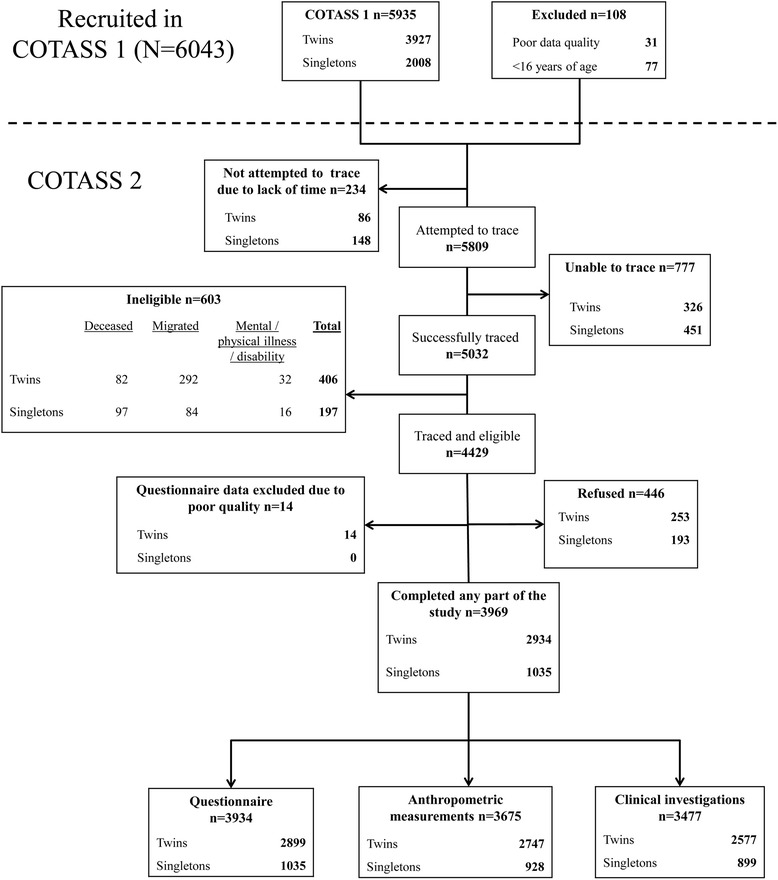
Flowchart of recruitment

The recruitment process is outlined in Fig. 1. We attempted to trace 5809 of 6043 COTASS-1 participants, and successfully traced 5032. Seven hundred seventy-seven could not be traced, and 603 of the traced participants were ineligible. Of the 4429 eligible participants, 446 refused to participate and 14 were excluded due to poor data quality. Thus, the final COTASS-2 sample consisted of 3969 participants who completed at least one study component. Ninety-three participants from the final COTASS-2 sample had been excluded from COTASS-1 due to poor data quality, or because they were younger than 16 at the time. Thus, the cohort size was 3876. The overall participation rate in COTASS-2 was 76.4, and 83.4% for twins and 61.8% for singletons. These participation rates excluded from the denominator COTASS-1 participants where recruitment was not attempted, those who were ineligible, and participants with poor quality data. Further excluding those not possible to trace produced an overall response rate of 89.9% (91.9% for twins, 84.6% for singletons). Detailed participation rates for each study component are presented as Additional file 1: Table S2. Participation rates were high, including 64.7% providing consent for their DNA to be stored for future genetic research.

### Sample characteristics

The COTASS-2 sample consisted of 73.4% (*n* = 2934) twins and 26.1% (*n* = 1035) singletons (Table 1). 57.6% were female, and ages ranged from 19 to 91 years, with a mean age of 42.8 years. The majority of the sample were married and lived in urban areas. The dominant ethnic group was Sinhalese (92.7%), and Buddhism was the most commonly reported religion (86.5%). Over half of the sample were in employment, and of those in employment, the most frequently reported occupation type was non-manual or skilled manual labour. Most COTASS-2 participants were educated beyond Grade 6, and 5% reported university education. Approximately 10% reported that making ends meet financially was “difficult” or “very difficult”, 3.9% reported hunger in the past 3 months because of insufficient money to buy food.

**Table 1:** Demographic and socio-economic characteristics of the COTASS-2 sample

	Total sample *N* = 3969	Twins *N* = 2934	Singletons *N* = 1035	Twins vs Singletons^a^
	%/mean (*n*)	%/mean (*n*)	%/mean (*n*)	Χ^2^/t-test *p*-value
Gender				0.001
Male	42.4 (1681)	43.9 (1289)	37.9 (392)	
Female	57.6 (2288)	56.1 (1645)	62.1 (643)	
Mean age	42.8 (3969)	39.9 (2934)	51.2 (1035)	<0.001
Age categories				<0.001
19–29	21.6 (865)	26.3 (772)	8.3 (86)	
30–39	25.8 (1025)	29.0 (852)	16.7 (173)	
40–49	21.0 (835)	20.7 (607)	22.0 (228)	
50–59	16.8 (667)	15.3 (449)	21.1 (218)	
60–69	9.6 (380)	5.9 (173)	20.0 (207)	
70+	5.1 (204)	2.8 (81)	11.9 (123)	
Marital status				<0.001
Married	72.2 (2838)	70.7 (2048)	76.5 (790)	
Widowed	6.3 (246)	3.8 (111)	13.1 (135)	
Divorced/Separated	2.1 (83)	1.8 (53)	2.9 (30)	
Never married	19.4 (763)	23.6 (685)	7.6 (78)	
Ethnicity				<0.001
Sinhala	92.7 (3647)	91.6 (2656)	95.7 (991)	
Tamil	3.1 (120)	3.5 (101)	1.8 (19)	
Muslim	3.8 (150)	4.5 (130)	1.9 (20)	
Other	0.4 (16)	0.4 (11)	0.5 (5)	
Religion				<0.001
Buddhist	86.5 (3401)	85.4 (2475)	89.5 (926)	
Hindu	1.6 (64)	2.0 (57)	0.7 (7)	
Islam	4.0 (159)	4.7 (137)	2.1 (22)	
Christian	7.9 (309)	7.9 (229)	7.7 (80)	
Urbanicity				<0.001
Urban	60.2 (2390)	59.9 (1758)	61.1 (632)	
Rural	13.4 (533)	13.0 (380)	14.8 (153)	
Mixed	20.8 (826)	19.9 (585)	23.3 (241)	
Outside Colombo	5.6 (221)	7.2 (212)	0.9 (9)	
Employment				<0.001
Employed	56.1 (2206)	59.6 (1727)	46.3 (479)	
At home	40.8 (1605)	36.9 (1070)	51.7 (535)	
Student	1.2 (48)	1.6 (46)	0.2 (2)	
Unemployed off sick	1.3 (52)	1.2 (35)	1.6 (17)	
Other	0.6 (23)	0.7 (21)	0.2 (2)	
Social class				<0.001
Managers/Professionals	8.1 (318)	9.3 (267)	4.9 (51)	
Non-manual workers/ Skilled manual workers	39.5 (1540)	41.7 (1197)	33.2 (343)	
Elementary occupations	8.3 (322)	8.3 (239)	8.0 (83)	
Not in employment	44.1 (1723)	40.7 (1167)	53.8 (556)	
Education				0.002
No education	1.8 (321)	1.8 (52)	1.6 (16)	
Grade 1–5	9.0 (2389)	9.2 (264)	8.6 (87)	
Grade 6-o/ls	42.9 (929)	40.9 (1174)	48.7 (490)	
Passed o/ls	18.5 (276)	18.8 (541)	17.6 (177)	
A/ls	22.3 (865)	23.3 (669)	19.5 (196)	
University or higher	5.0 (193)	5.4 (156)	3.7 (37)	
Other	0.5 (20)	0.6 (17)	0.3 (3)	
Financial strain				<0.001
Living comfortably	9.3 (365)	9.4 (273)	8.9 (92)	
Doing alright	66.5 (2616)	68.3 (1979)	61.5 (637)	
Just about getting by	13.9 (547)	13.4 (388)	15.4 (159)	
Difficult to make ends meet	7.2 (284)	6.3 (182)	9.9 (102)	
Very difficult to make ends meet	3.1 (121)	2.6 (76)	4.3 (45)	
Hungry due to lack of money				<0.001
No	96.1 (3779)	97.2 (2815)	93.1 (964)	
Yes	3.9 (153)	2.8 (82)	6.9 (71)	

Due to incomplete questionnaire data, the numbers do not add up to totals presented beyond gender and age

^a^Analyses account for clustering by twins

Twins and singletons differed on several socio-demographic and socio-economic characteristics (Table 2). Notably, singletons were older and a higher proportion were women, married or widowed, Colombo residents, of Sinhalese ethnicity and of Buddhist religious affiliation. Singletons had lower socio-economic status: they were less likely to be employed or in managerial/professional occupational positions, reported lower education, and greater financial strain. Post-hoc analyses tested socio-demographic differences between twins and singletons, by including age, gender, marital status, and ethnicity simultaneously in a logistic regression model (religion substantially overlapped with ethnicity and was excluded to avoid collinearity; analyses not shown). These variables were found to independently distinguish twins from singletons. Further post-hoc regression tests indicated that differences in employment, occupation and education differences were driven by age. However, singletons remained at greater risk of reporting making ends meet “difficult” (*p* = 0.035), and hunger due to insufficient money (*p* < 0.001). Zygosity characteristics presented in Table 2 are in line with the usual distribution seen in population studies: slightly more MZ pairs and slightly more female participants, with opposite sex pairs being the biggest group.

**Table 2:** Twins participating in COTASS-2

Zygosity	Number of Individual	Number of Twin Families
Monozygotic Males (MZM)	533	295
Dizygotic Males (DZM)	366	215
Monozygotic Females (MZF)	730	397
Dizygotic females (DZF)	485	281
Dizygotic Opposite Sex (DZOS)	809	467
Triplets	11	5
Zygosity missing	1	–

Number of individuals in the twin groups is not twice the number of families due to missing data. Actual numbers of ‘complete pairs’ are not given since those differ per variable

### Systematic differences due to loss to follow-up

Compared to those lost to follow-up (*n* = 2059), a greater proportion of the followed-up participants in COTASS-2 (*n* = 3876) were twins, female, of younger age, of Sinhalese ethnicity, and residing in semi-urban areas. Those who were followed-up also had slightly poorer self-rated health; post-hoc analyses indicated that gender drove this association, as more women reported fair or poor health (not shown). There were no attritional differences according to marital status, socio-economic status or depression (Table 3).

**Table 3:** Characteristics of respondents not followed-up and followed-up in COTASS-2, using data from COTASS-1

	Lost to follow up (COTASS 1 only, *N* = 2059)	Followed-up (COTASS1 + 2, *N* = 3876)^a^	Lost to follow-up VS followed up^b^
	% /mean (*n*)	% /mean (*n*)	*p*-value Χ^2^ / t-test
Twin or singleton			<0.001
Twin	52.4 (1078)	73.5 (2849)	
Singleton	47.6 (981)	26.5 (1027)	
Gender			<0.001
Male	52.5 (1082)	42.1 (1633)	
Female	47.5 (977)	57.9 (2243)	
Mean age (years)	38.0 (2055)	36.7 (3876)	<0.001
Age (years)			<0.001
16–24	25.1 (515)	23.7 (918)	
25–34	24.0 (494)	26.4 (1024)	
35–44	18.4 (379)	20.6 (798)	
45–54	15.0 (308)	16.2 (627)	
55–64	10.0 (205)	9.0 (349)	
65–74	5.3 (109)	3.5 (136)	
75 or over	2.2 (45)	0.6 (24)	
Marital status			0.114
Married	58.0 (1194)	61.4 (2378)	
Widowed	5.1 (105)	4.8 (185)	
Divorced/Separated	1.6 (32)	1.4 (53)	
Never married	35.3 (727)	32.4 (1255)	
Ethnicity			0.001
Sinhala	90.4 (1862)	93.4 (3619)	
Tamil	3.1 (63)	2.6 (102)	
Muslim	6.1 (126)	3.7 (145)	
Other	0.4 (8)	0.3 (10)	
Urbanicity			<0.001
Semi-urban	56.8 (1170)	62.9 (2436)	
Urban	43.2 (889)	37.1 (1438)	
Employment			0.118
Not in employment	39.4 (809)	41.8 (1617)	
Student	8.2 (169)	8.0 (308)	
Part-time employment	13.0 (267)	14.2 (549)	
Full-time employment	39.3 (806)	36.1 (1396)	
Social class			0.079
Rich	30.5 (252)	25.9 (385)	
Middle class	33.3 (276)	34.9 (518)	
Poor	36.2 (299)	39.2 (583)	
Financial strain			0.177
Living comfortably	8.9 (183)	7.9 (304)	
Doing alright	64.1 (1316)	64.7 (2504)	
Just about getting by	13.0 (267)	14.6 (563)	
Difficult to make ends meet	8.9 (182)	8.7 (336)	
Very difficult to make ends meet	5.2 (106)	4.2 (162)	
Hungry due to lack of money			0.653
No	96.3 (1978)	96.0 (3716)	
Yes	3.7 (77)	4.0 (155)	
Self-rated health			0.032
Good, very good or excellent	57.5 (1179)	54.6 (2109)	
Fair or poor	42.5 (870)	45.4 (1756)	
Depressive episode (lifetime)^c^			0.750
No	94.6 (1945)	94.4 (3653)	
Yes	5.4 (110)	5.6 (215)	

^a^Followed-up respondents represent the cohort with data at both COTASS 1 and 2; COTASS 2 respondents which were excluded from COTASS 1 could not be included in these analyses (*n* = 93)

^b^Analyses account for clustering by twins

^c^Estimated using the Composite International Diagnostic Interview

### Prevalence and distribution of mental disorders

Table 4 presents prevalence estimates of mental disorder and associations with demographic characteristics, separately by twin and singleton status. Informed by the differences observed Table 1, associations adjusted for age, sex, education marital status and financial strain. Twins and singletons reported similar levels of depressive disorder and PTSD. Just under 4% of twins and singletons met the DSM-IV criteria for major depressive episode in the past year using the CIDI, and approximately 5% reported met the DSM criteria for PTDS. Hazardous alcohol use was highly prevalent in male twins and singletons (28.1 and 30.9%, respectively). Twins had lower estimates depressive and anxiety symptoms compared to singletons (5.9% vs. 9.8%, and 3.6% vs. 5.1%, respectively). Twins and singletons’ mean scores of depressive symptoms and anxiety symptoms were, respectively, 4.4 and 6.0 on the BDI-II, and 1.7 and 2.2 on the GAD-7 (not shown).

**Table 4:** Prevalence and adjusted associations of mental disorders with sociodemographic indicators in twins and singletons

	Total sample	Gender		Education		Age		
		Male	Female	Higher	Lower	19–34	35–54	55+
Twins	(*N* = 2934)	(*n* = 1289)	(*n* = 1645)	(*n* = 960)	(*n* = 1923)	(*n* = 1197)	(*n* = 1302)	(*n* = 435)
Depressive disorder (*N* = 2899)								
% (n)	3.8 (110)	3.1 (39)	4.4 (71)	3.6 (35)	3.9 (75)	4.1 (48)	4.0 (52)	2.3 (10)
AOR (95% CI)		ref.	1.3 (0.9–2.0)	ref.	1.1 (0.7–1.7)	ref.	0.9 (0.6–1.4)	0.3* (0.1–0.8)
Depressive symptoms (*N* = 2883)								
% (n)	5.9 (170)	4.1 (52)	7.3 (118)	3.6 (34)	7.1 (136)	5.5 (65)	5.9 (76)	6.8 (29)
AOR (95% CI)		ref.	1.7** (1.2–2.5)	ref.	2.0** (1.3–3.1)	ref.	0.9 (0.6–1.4)	0.8 (0.4–1.4)
Generalised anxiety disorder (*N* = 2893)								
% (n)	3.6 (103)	2.5 (32)	4.4 (71)	2.7 (26)	4.0 (77)	3.6 (42)	3.6 (46)	3.5 (15)
AOR (95% CI)		ref.	1.7* (1.1–2.6)	ref.	1.5 (0.9–2.6)	ref.	0.9 (0.5–1.5)	0.6 (0.3–1.5)
PTSD (*N* = 2896)								
% (n)	4.5 (131)	3.6 (46)	5.2 (85)	2.7 (26)	5.4 (104)	4.1 (49)	5.1 (65)	4.0 (17)
AOR (95% CI)		ref.	1.4 (0.9–2.0)	ref.	2.1** (1.3–3.4)	ref.	1.0 (0.7–1.6)	0.4* (0.4–0.8)
*Men only* ^*a*^	*(N = 1289)*	*–*	*–*	*(n = 400)*	*(n = 861)*	*(n = 557)*	*(n = 562)*	*(n = 170)*
Hazardous alcohol use (*N* = 1658)								
% (n)	28.1 (356)	–	–	18.0 (72)	32.6 (280)	20.9 (115)	36.7 (202)	23.2 (39)
AOR (95% CI)		–	–	ref.	1.9*** (1.4–2.6)	ref.	1.7** (1.2–2.4)	1.0 (0.5–1.3)
Singletons	(*N* = 1035)	(*n* = 392)	(*n* = 643)	(*n* = 245)	(*n* = 787)	(*n* = 146)	(*n* = 450)	(*n* = 439)
Depressive disorder (*N* = 1035)								
% (n)	3.9 (40)	2.6 (10)	4.7 (30)	4.9 (12)	3.6 (28)	7.5 (11)	4.0 (18)	2.5 (11)
AOR (95% CI)		ref.	1.7 (0.8–3.6)	ref.	0.7 (0.4–1.5)	ref.	0.5 (0.2–1.1)	0.3** (0.1–0.7)
Depressive symptoms (*N* = 1030)								
% (n)	9.8 (101)	6.7 (26)	11.7 (75)	5.7 (14)	11.0 (86)	6.2 (9)	10.5 (47)	10.3 (45)
AOR (95% CI)		ref.	1.6 (0.98–2.6)	ref.	1.7 (0.9–3.1)	ref.	1.9 (0.8–4.5)	1.6 (0.7–3.6)
Generalised anxiety disorder (*N* = 1034)								
% (n)	5.1 (53)	4.1 (16)	5.8 (37)	2.9 (7)	5.9 (46)	4.8 (7)	6.4 (29)	3.9 (17)
AOR (95% CI)		ref.	1.3 (0.7–2.6)	ref.	2.3 (0.99–5.3)	ref.	1.7 (0.6–4.7)	0.9 (0.3–2.6)
PTSD (*N* = 1035)								
% (n)	5.4 (56)	4.1 (16)	6.2 (40)	4.5 (11)	5.7 (45)	5.5 (8)	7.3 (33)	3.4 (15)
AOR (95% CI)		ref.	1.5 (0.8–2.7)	ref.	1.3 (0.7–2.7)	ref.	1.3 (0.5–3.2)	0.6 (0.2–1.5)
*Men only* ^*a*^	*(n = 392)*	*–*	*–*	*(n = 101)*	*(n = 290)*	*(n = 65)*	*(n = 143)*	*(n = 184)*
Hazardous alcohol use (*N* = 391)								
% (n)	30.9 (121)	–	–	24.0 (24)	33.4 (97)	33.8 (22)	44.8 (64)	19.1 (35)
AOR (95% CI)		–	–	ref.	1.9* (1.1–3.3)	ref.	1.9 (0.9–3.9)	0.5 (0.2–1.1)

Odds ratios adjust for age, gender, education, marital status, ethnicity and financial strain

*AOR* Adjusted odds ratio, *CI* confidence interval; *PTSD* Post-Traumatic Stress Disorder, *DSM* Diagnostic and Statistical Manual of Mental Disorders

*p* < 0.05, ** *p* < 0.01, ***p < 0.001 ^a^Limited to men only, due to very low prevalence in female group (0.04%, *n* = 1); AORs adjusted for education and age only

The socio-demographic distributions of mental disorders followed similar patterns in twins and singletons. Mental disorders were more generally more common in women, and among those with lower educational attainment. Regression analyses controlling for demographic characteristics indicated few statistically significant differences. These included gender differences for depressive and anxiety symptoms in twins, and educational differences in hazardous alcohol use in twins and singletons, and depressive symptoms and PTSD in twins. Depressive disorder and PTSD was less common in the older age group (55+) compared to those aged 19–34. Depressive symptoms and anxiety symptoms did not vary by age, while hazardous alcohol use peaked in men aged 35–54 years.

### Prevalence and distribution of metabolic syndrome

Table 5 presents prevalence distributions of cardiometabolic risk factors, and adjusted associations with demographic characteristics for twins and singletons. A third of the twin sample and nearly half of the singletons met the metS criteria (Table 5). The prevalence was slightly higher according to the NCEP ATP III definition in both samples. There were substantial differences in metS by gender. Approximately 34% of female twins, and 55% of female singletons met the metS criteria; whereas 26 and 36% of men in the respective samples met these criteria. These differences were driven by a high proportion of women meeting the gender-specific criteria for central obesity: over 78% of female twins and singletons had waist circumferences ≥80 cm while only 35% of male twins and 42% of male singletons were above the ≥90 cm threshold. This put female twins and singletons at 7- and 12-fold increased odds of central obesity, respectively. In comparison, the gender differences in the BMI indicator of overweight by were not as pronounced (AORs ≥ 2.1; Table 5). The prevalence of individual metS components ranged from 25 to 59% in twins and 30–71% in singletons. Aside from waist circumference, raised fasting glucose or diabetes was the most common metS component, with 38% of twins and 58% of singletons above the threshold. Raised triglyceride levels was the least common component in both samples, but still over a quarter of twins and singletons were at elevated risk. In both samples, women were at three times’ increased odds of reduced HDL-cholesterol, while men were at greater odds of raised triglyceride levels. The blood pressure component and raised fasting glucose or diabetes component did not vary by gender. There were few cardiometabolic risk differences by education; only BMI and waist circumference was lower among those with lower education (differences were statistically significant in twins, and marginally significant in singletons). MetS components were more common in the older age groups, and those aged 55+ were at particularly elevated odds of raised blood pressure (AOR ≥ 10.5) and raised fasting glucose or diabetes (AOR ≥ 2.8), compared to those aged 19–34. Age distributions of cardiometabolic risk were similar between twins and singletons, with the exception of central obesity and BMI. These indicated that younger (19–34) and middle-age (35–54) singletons had higher BMI and waist circumference than equivalent age groups in twins, whilst prevalence estimates in the 55+ age groups were comparable.

**Table 5:** Prevalence and adjusted associations of cardiometabolic risk indicators with sociodemographic indicators in twins and singletons

	Total sample	Gender		Education		Age		
		Male	Female	Higher	Lower	19–34	35–54	55+
Twins	(*N* = 2934)	(*n* = 1289)	(*n* = 1645)	(*n* = 960)	(*n* = 1923)	(*n* = 1197)	(*n* = 1302)	(*n* = 435)
MetS: IDF (*N* = 2553)								
% (n)	27.4 (700)	19.9 (229)	33.6 (471)	25.0 (200)	28.7 (491)	13.8 (142)	33.7 (388)	45.5 (170)
AOR (95% CI)		ref.	2.0*** (1.6–2.4)	ref.	0.8 (0.7–1.0)	ref.	2.6*** (2.0–3.4)	4.3*** (3.1–5.9)
MetS: NCEP ATP III (*N* = 2553)								
% (n)	30.6 (780)	25.9 (298)	34.4 (482)	27.6 (221)	32.1 (549)	16.1 (166)	37.0 (425)	50.5 (189)
AOR (95% CI)		ref.	1.4** (1.2–1.7)	ref.	0.8 (0.7–1.0)	ref.	2.5*** (1.9–3.1)	4.3*** (3.1–5.8)
BMI ≥23 (*N* = 2738)								
% (n)	52.3 (1431)	45.7 (559)	57.6 (872)	55.2 (487)	50.9 (920)	45.5 (502)	57.3 (703)	55.5 (226)
AOR (95% CI)		ref.	1.5*** (1.3–1.8)	ref.	0.7*** (0.6–0.8)	ref.	1.4** (1.1–1.7)	1.3* (1.0–1.8)
Central obesity (*N* = 2743)								
% (n)	59.2 (1623)	34.8 (425)	78.8 (1198)	62.9 (555)	57.7 (1045)	48.4 (535)	65.9 (810)	68.0 (278)
AOR (95% CI)		ref.	7.4*** (6.0–9.0)	ref.	0.5*** (0.4–0.7)	ref.	1.8*** (1.4–2.3)	2.0*** (1.5–2.8)
Raised triglycerides (*N* = 2577)								
% (n)	25.0 (644)	35.6 (413)	16.3 (231)	24.4 (197)	25.5 (439)	21.1 (220)	28.6 (331)	24.7 (93)
AOR (95% CI)		ref.	0.3*** (0.3–0.4)	ref.	0.9 (0.7–1.1)	ref.	1.5** (1.2–1.9)	1.4 (0.8–1.6)
Low HDL cholesterol (*N* = 2577)								
% (n)	32.3 (832)	20.2 (234)	42.2 (598)	30.8 (249)	33.0 (569)	34.7 (362)	31.5 (364)	28.2 (106)
AOR (95% CI)		ref.	2.9*** (2.4–3.5)	ref.	1.2 (0.9–1.5)	ref.	0.7** (0.5–0.8)	0.5*** (0.4–0.8)
Raised BP /hypertensive tx (*N* = 2747)								
% (n)	27.4 (754)	28.0 (343)	27.0 (411)	19.9 (176)	31.2 (565)	11.4 (126)	30.3 (373)	62.2 (255)
AOR (95% CI)		ref.	0.8 (0.7–1.0)	ref.	1.1 (0.9–1.4)	ref.	3.0*** (2.3–3.9)	10.5***(7.6–14.5)
Raised fasting glucose/diabetes (*N* = 2597)								
% (n)	38.2 (992)	38.1 (444)	38.3 (548)	34.1 (279)	40.1 (695)	20.3 (212)	46.4 (541)	61.8 (239)
AOR (95% CI)		ref.	0.9 (0.8–1.1)	ref.	0.9 (0.7–1.1)	ref.	3.0*** (2.4–3.7)	5.9*** (4.4–7.8)
Singletons	(*N* = 1035)	(*n* = 392)	(*n* = 643)	(*n* = 245)	(*n* = 787)	(*n* = 146)	(*n* = 450)	(*n* = 439)
MetS: IDF (*N* = 891)								
% (n)	44.6 (397)	28.3 (98)	54.9 (299)	41.2 (89)	45.4 (305)	27.9 (34)	43.1 (175)	51.8 (188)
AOR (95% CI)		ref.	3.5*** (2.5–4.8)	ref.	1.0 (0.7–1.4)	ref.	1.6 (1.0–2.5)	2.6*** (1.6–4.3)
MetS: NCEP ATP III (*N* = 891)								
% (n)	48.6 (433)	36.4 (126)	56.3 (307)	45.8 (99)	49.3 (331)	29.5 (36)	46.3 (188)	57.6 (209)
AOR (95% CI)		ref.	2.6*** (1.9–3.6)	ref.	1.0 (0.7–1.4)	ref.	1.7* (1.0–2.7)	3.0*** (1.9–4.9)
BMI ≥23 (*N* = 925)								
% (n)	61.0 (564)	49.7 (177)	68.0 (387)	65.0 (147)	59.6 (415)	63.0 (80)	68.1 (282)	52.6 (202)
AOR (95% CI)		ref.	2.1*** (1.6–2.8)	ref.	0.8 (0.6–1.1)	ref.	1.0 (0.6–1.5)	0.6* (0.4–0.9)
Central obesity (*N* = 926)								
% (n)	71.2 (659)	41.7 (149)	89.6 (510)	72.1 (163)	70.7 (493)	63.8 (81)	75.4 (313)	69.0 (265)
AOR (95% CI)		ref.	12.4*** (8.6–18.0)	ref.	0.7 (0.5–1.1)	ref.	1.1 (0.6–1.9)	1.0 (0.6–1.7)
Raised triglycerides (*N* = 899)								
% (n)	29.6 (266)	37.2 (130)	24.7 (136)	32.7 (71)	28.7 (195)	31.5 (39)	31.0 (127)	27.4 (100)
AOR (95% CI)		ref.	0.6*** (0.4–0.8)	ref.	0.8 (0.6–1.1)	ref.	1.0 (0.6–1.6)	0.8 (0.6–1.2)
Low HDL cholesterol (*N* = 899)								
% (n)	39.9 (359)	24.9 (87)	49.5 (272)	39.2 (85)	39.9 (271)	38.7 (48)	44.6 (183)	35.1 (128)
AOR (95% CI)		ref.	2.9*** (2.2–4.0)	ref.	1.0 (0.7–1.4)	ref.	1.1 (0.7–1.8)	0.8 (0.5–1.3)
Raised BP /hypertensive tx (*N* = 928)								
% (n)	46.6 (432)	48.7 (175)	45.2 (257)	40.7 (92)	48.5 (339)	12.5 (16)	33.0 (137)	72.5 (279)
AOR (95% CI)		ref.	0.9 (0.7–1.3)	ref.	1.0 (0.7–1.4)	ref.	3.3*** (1.8–6.1)	16.6***(8.9–30.9)
Raised fasting glucose/diabetes (*N* = 923)								
% (n)	56.7 (523)	54.9 (196)	57.8 (327)	52.3 (115)	57.9 (405)	39.5 (49)	51.2 (212)	68.1 (262)
AOR (95% CI)		ref.	1.3 (0.97–1.7)	ref.	1.2 (0.8–1.6)	ref.	1.2 (0.8–1.9)	2.8*** (1.8–4.4)

Odds ratios adjust for age, gender, education, marital status, ethnicity and financial strain

*AOR* Adjusted odds ratio, *CI* confidence interval, *MetS* metabolic syndrome, *IDF* International Diabetes Federation criteria, *NCEP ATP III* revised National Cholesterol Education Programme Adult Treatment Panel criteria, *BMI* Body-Mass Index, *BP* blood pressure

* *p* < 0.05, ** *p* < 0.01, ***p < 0.001

## Discussion

COTASS-2 is a well-phenotyped, genetically-sensitive, epidemiological sample within a middle income country setting. As such it provides a unique resource to explore the associations between the underlying constituents of metS and wider risk factors for CVD and diabetes and mental disorders. It also provides a basis for a better understanding of the inter-relationships between mental and physical health more broadly.

### Prevalence of mental disorders

Our sample had a generally low prevalence of most mental disorders. 3.8% of twins and 3.9% of singletons met the criteria for major depressive disorder (DSM-IV), and 3.6% of twins and 5.1% of singletons reported moderate or severe anxiety symptoms (GAD-7 ≥ 10). Whilst the prevalence of major depressive disorder are in line with previous studies in Colombo (6.4%) and other South Asian populations (4.5% in India and 4.0% in Indian minority groups in Singapore [45, 52, 53]), most international studies report higher estimates (e.g. 8.3% in USA, and 5.5 and 5.9% in high-income countries and LMIC, respectively [52]). The estimates of anxiety symptoms were low within an international context (e.g. 8.2% (GAD-7 ≥ 8) in North America, 5.1% (GAD-7 ≥ 10) in Europe, and 11.2% (GAD-7 ≥ 10) in Pakistan [54]). In contrast, the prevalence of PTSD symptoms of 4.5% in twins and 5.4% in singletons was broadly consistent with international research (3.5% in USA [55], 5.5% in UK [56]). Virtually no women reported hazardous alcohol use, while approximately one third of men were hazardous drinkers. The prevalence is high compared to Asian groups in the UK [57], and is consistent with alcohol use being recognised as an increasingly common problem in Sri Lanka [58].

### Prevalence of metabolic syndrome and its constituent components

By contrast with mental disorders, we report a very high prevalence of metS (IDF: 27.4% in twins, 44.6% in singletons; NCEP ATP III: 30.6% in twins, 48.6% in singletons). These findings are consistent with previously observed estimates in urban areas of Sri Lanka (IDF: 34.8% [59]; IDF: 38.9%, revised NCEP ATP III: 46.1% in adults aged 35–65 [60]). Gender-specific prevalence estimates are also comparable to those found in the Sri Lankan population (COTASS-2: 19.9–28.3% and 33.6–54.9%; Sri Lanka Diabetes and Cardiovascular Study: 24.3 and 40.8%; in men and women, respectively [59]). This suggests that future analyses of this sample are likely to be broadly generalisable for Sri Lanka. More broadly, urban household studies in South Asia typically find prevalence estimates that are comparable to the singleton estimates in COTASS-2, both according to IDF criteria (35% in Pakistan) [61], and various versions of the NCEP ATP III criteria (41–49% in India and Pakistan) [61–63]. In wider international comparisons, the COTASS-2 estimates are in line with the US general population (NCEP ATP III: 33% [64]), South Asian immigrant adults in the US (IDF: 29.7% [65]; IDF: 38.2%, revised NCEP ATP III: 32.7% [66]), and men of South Asian ethnicity aged 40–69 in the UK (IDF: 44.5%) [67].

The prevalence of metS increased with age, consistent with the literature [59, 60, 64, 68], but few differences were observed by levels of education. While US and other Western studies indicate higher prevalence of metS in groups with low education, the Sri Lanka Diabetes and Cardiovascular Study, carried out in 2005–2006, found that higher education groups were more likely to meet the criteria for metS [59, 69]. Given that the association between metS and socioeconomic status seems to be heavily influenced by health behaviours [70], the findings from our more recent study may indicate a shift of socioeconomic distributions of health behaviours in Sri Lanka towards those observed in Western populations.

The gender-specific distributions of metS components were broadly consistent with previous research [9, 60, 66, 71, 72]. Women were more likely to have low HDL cholesterol and higher waist circumference, while men more likely to have raised triglyceride levels, and there were no differences in hypertension or raised blood glucose levels. The most common metS components were central obesity and raised fasting blood glucose or diabetes. Central obesity was a more prominent feature compared to other Sri Lankan studies where the most prevalent components were hypertension [59] and fasting blood glucose [60]. The variability of individual metS components within Sri Lanka may be indicative of a lack of consistent clustering of metS components in the population.

The high estimates of central obesity are consistent with the increasing prevalence of obesity in Sri Lanka which has been described as reaching epidemic proportions [10, 72]. Central obesity was particularly prevalent among women, and this gender discrepancy was much greater than the differences in overweight defined by BMI. However, the very high prevalence of central obesity (78.8% in female twins; 89.6% in female singletons) was not consistent with the prevalence of other metS components (<60%). This may suggest that the 80 cm cut-off of waist circumference overestimates cardiometabolic risk for Sri Lankan women. The observation that twins were less likely than singletons to be overweight, particularly in younger age groups, could be an effect of twins’ lower birth weight, affecting weight in adulthood [73].

Raised blood glucose levels were alarmingly high at 38.2% of twins and 56.7% of singletons. This was considerably higher than observed in studies in the US and India [74, 75], and substantially higher than previously observed rates of dysglycaemia in urban areas of the Sri Lanka (30.0%) [7]. The prevalence was particularly high among older adults, but also elevated in younger adults. These findings re-affirm concerns of a diabetes epidemic in Sri Lanka [76].

### Limitations

Our original sample had an exceptionally high participation rate (91% for twins and 87% for singletons). The present study achieved good follow up rates, particularly for twins (83.4%), where participants usually had contact details for their co-twins. There is always a risk of bias caused by non-participation, which is particularly important for mental disorders, which have a critical impact on participation [77]. However, unlike many surveys we did not find that depression influenced participation, suggesting that attrition will have minimal influence on analyses examining mental health outcomes. Furthermore, given that neither depression nor self-rated health determined follow-up, it is unlikely that loss to follow-up will influence the findings. As this sample had completed a psychiatric interview during the previous wave of data collection (COTASS-1), it is possible that individuals identified as having a mental disorder followed our advice to seek treatment, which caused a reduction in prevalence of mental disorders in COTASS-2. However, given that this was merely a recommendation rather than a formal intervention, it is unlikely that a high proportion advised to consult did so. We therefore believe that this did not have a major impact on prevalence. Furthermore, the lifetime depressive disorder outcome captured by the CIDI will not have been affected by ascertaining mental disorder at a previous timepoint.

It is possible that twins differ from non-twins in ways that affect health and limit the interpretation of our prevalence estimates. Our analyses presented the results by twins and singletons, and showed some modest differences in mental health, whilst, singletons were at greater cardiometabolic risk compared to twins. Twins and singletons’ sociodemographic distributions of mental disorders and cardiometabolic risk were similar.

In terms of data collection, due to the multicomponent nature of the study with separate specialist data collection teams, it was not always possible to collect data from all components simultaneously for each participant. In addition, biospecimens were collected early in the morning whereas questionnaire data and anthropometric measurements could be collected at any time during the day when participants were available.

## Conclusions

We successfully followed up the previous cohort of twins and singletons. The initial findings indicate that an exceptionally high proportion of the singleton population we studied have metS – a finding of profound importance for public health planning, which suggests that over the next decades its impact will be felt in terms of increasing prevalence of diabetes and cardiovascular disease. Our findings indicate the need for population level preventive approaches particularly targeting diet and exercise. Future papers will describe in greater depth the association between metS variables, their genetic architecture and association with mental health.

## Additional file

Additional file 1: Table S1. Description of measures included in the questionnaire of CoTaSS 2. **Table S2.** Number of participants for each component and respective response rates. (DOCX 43 kb)
