# Supplementary material for: The Colombo Twin and Singleton Follow-up Study: a population based twin study of psychiatric disorders and metabolic syndrome in Sri Lanka
Source: BMC Public Health. 2018 Jan 17;18:145. doi: 10.1186/s12889-017-4992-2 (PMC5773033; doi:10.1186/s12889-017-4992-2)
Supplement: Additional file 1: Table S1. — Description of measures included in the questionnaire of CoTaSS 2. Table S2. Number of participants for each component and respective response rates. (DOCX 43 kb) [file 12889_2017_4992_MOESM1_ESM.docx]

Additional file 1

Table S1 Description of measures included in the questionnaire of CoTaSS 2

| **Type of measure** | **Description of measure** |
| --- | --- |
| Sociodemographic measures | Sociodemographic information (e.g. gender, age, ethnicity), socio-economic status (e.g. occupation education), household composition, and housing quality [23]. |
| Depression | 12-month version of section E of the WHO-Composite International Diagnostic Interview [26]. |
|  | Severity of depression measured using the Beck Depression Inventory [27]. |
| Post-traumatic stress disorder | 17 items checklist which corresponds to the key symptoms of PTSD [28]. |
| Anxiety | Generalised Anxiety Disorder questionnaire. Screening and measuring severity of generalised anxiety disorder (civilian version) [29]. |
| Somatic symptoms | Bradford Somatic Inventory. Screening for somatic symptoms associated with anxiety and depression [30]. |
| Fatigue | Chalder Fatigue Scale. Measures the severity and extent of fatigue [31]. |
| Measure of social support | Multi-dimensional support scale questionnaire [41]. |
| Tobacco use | Tobacco Use questionnaire [38]. |
| Alcohol use | Alcohol Use Disorders Identification Test [39]. |
| Stressful life events | A list of threatening experiences [40]. |
| Eating disorders | The Dutch Eating Behaviour Questionnaire [32] and the Three-Factor Eating Questionnaire [33] were adopted for this eating disorders questionnaire. |
| Health and well-being | Short Form 36 Health Survey Questionnaire [34]. |
| Physical activity | International Physical Activity Questionnaire (IPAQ), an internationally validated screen of physical activity for the past 7 days [36]. |
| Sleep | Pittsburgh sleep quality index. Assesses sleep quality over the period of a month [35]. |
| Zygosity determination | Adapted from zygosity determination questionnaire designed by Ooki, Yamada and Asaka during first CoTaSS study [42]. |
| Closeness of twins | Closeness of twins questionnaire based on items from questionnaires designed by Loehlin and Nichols (1976) [43], and Kendler and Gardner (1998) [44]. |
| Diet and frequency of food groups | A adapted questionnaire to measure the frequency of consumption of a comprehensive list of food groups in Sri Lanka, designed to assess dietary habits [37]. |
| Physical illnesses | A checklist of physical illnesses designed by Institute for Research and Development. |

Table S2 Number of participants for each component and respective response rates

|  | **Total sample** | | **Twins** | | **Singletons** | |
| --- | --- | --- | --- | --- | --- | --- |
| **Type of data collected** | **N** | **Participation rate**  **(N=5192)*** | **N** | **Participation rate**  **(N=3518)*** | **N** | **Participation rate**  **(N=1674)*** |
| Any completed component | 3969 | 76.4% | 2934 | 83.4% | 1035 | 61.8% |
| All components completed | 3420 | 65.9% | 2527 | 71.8% | 893 | 53.3% |
| Questionnaires | 3934 | 75.8% | 2899 | 82.4% | 1035 | 61.8% |
| Anthropometric Measurements | 3675 | 70.8% | 2747 | 78.1% | 928 | 55.4% |
| Clinical investigations | 3476 | 66.9% | 2577 | 73.3% | 899 | 53.7% |
| Whole blood for clinical investigations & biobanking | 3476 | 66.9% | 2577 | 73.3% | 899 | 53.7% |
| Urine for clinical investigations | 3470 | 66.8% | 2571 | 73.1% | 899 | 53.7% |
| Serum for biobanking | 3476 | 66.9% | 2577 | 73.3% | 899 | 53.7% |
| DNA for biobanking | 3360 | 64.7% | 2488 | 70.7% | 872 | 52.1% |

***** Denominators include those who could not be traced, and exclude COTASS 1 participants who were not attempted to contact, ineligible or excluded due to poor data quality, (see Figure 1).

,
